# AC Kelvin Probe Force Microscopy Enables Charge Mapping in Water

**DOI:** 10.1021/acsnano.2c07121

**Published:** 2022-10-10

**Authors:** Thomas Hackl, Georg Schitter, Patrick Mesquida

**Affiliations:** † Automation and Control Institute (ACIN), TU Wien, Gusshausstrasse 27-29, A-1040Vienna, Austria; ‡ Department of Physics, King’s College London, Strand, LondonWC2R 2LS, United Kingdom

**Keywords:** AC-KPFM, surface charge, atomic force microscopy, microcontact printing, self-assembled monolayer, solid−liquid interface

## Abstract

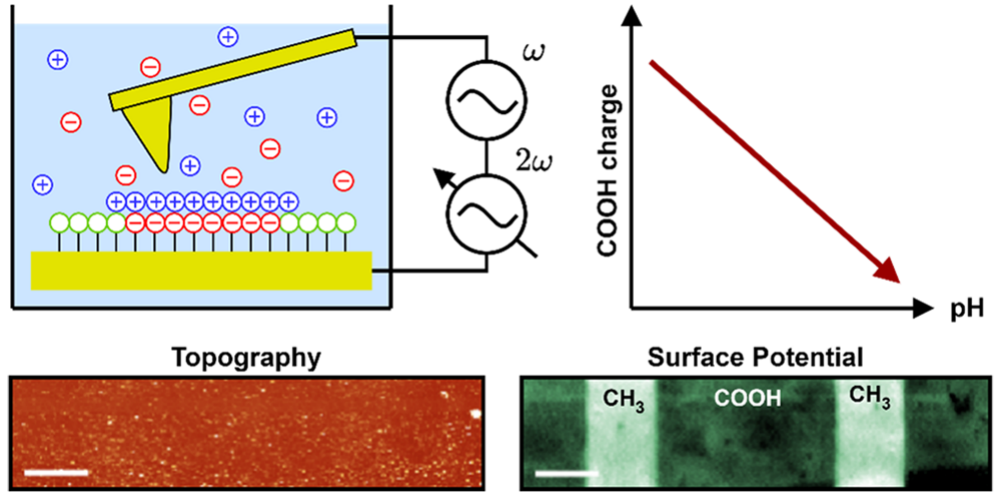

Mapping charged chemical groups at the solid–liquid interface is important in many areas, ranging from colloidal systems to biomolecular interactions. However, classical methods to measure surface charges either lack spatial resolution or—like Kelvin-probe force microscopy (KPFM)—cannot be applied in aqueous solutions because a DC bias voltage is used. Here, we show that using AC Kelvin probe force microscopy (AC-KPFM), in which the DC bias is replaced with an AC voltage of sufficiently high frequency, the surface potential of spatially fixated, charged surface groups can be mapped in aqueous solution. We demonstrate this with micropatterned, functionalized alkanethiol layers which expose ionized amino- and carboxy-groups. These groups are representative of the charged groups of most biomolecules such as proteins. By adjusting the pH of the solution, the charge of the groups was reversibly altered, demonstrating the electrostatic nature of the measured signal. The influence of the electric double layer (EDL) on the measurement is discussed, and we, furthermore, show how charged, micropatterned layers can be used to spatially direct the deposition of nanoparticles of opposite charge.

Surface charges or the related zeta-potentials in water are usually determined by dynamic light scattering or streaming-potential measurements.^1^ However, these are indirect methods in that they rely on a hydrodynamic model, they are only applicable to dispersed particles or porous materials, and they do not provide spatial resolution. The only direct (in the sense that actual electrostatic forces are measured) and spatially resolved techniques for mapping surface charges are based on atomic force microscopy (AFM), where a tiny tip raster-scans over the surface of a sample. The classical example is Kelvin probe force microscopy (KPFM), which has been employed widely in materials and semiconductor science since the early 1990s.^2^ KPFM comes in many variations, but, essentially, the local, electrostatic force is detected by a conductive tip to which a bias voltage is applied, while the tip is scanned very close (a few tens of nm at most) to the sample.

The oldest and still most widely used implementation of KPFM is amplitude-modulated (AM)-KPFM,^3^ which is an option available with most commercial AFM instruments. By modulating the bias voltage, the electrostatic force between tip and sample is nullified by a controller, thereby giving out the local surface potential, φ = φ(x,y), a quantity closely related to the charge distribution on the sample surface. However, classical AM-KPFM and the more recent method of frequency-modulated (FM)-KPFM^4^ use a DC-bias as a modulation signal, which precludes their use in aqueous solutions. This significantly limits the application of KPFM to biomolecular or similar soft-matter structures. Measurements on dried samples are possible, but they can only give a tentative indication of its electrostatic properties in liquid.^5−7^

The DC-bias or, more generally, any low-frequency voltage applied between tip and sample leads to an unwanted voltage breakdown in aqueous solutions.^8^ This is due to the presence of polar water molecules and highly mobile electrolyte ions in water and their response to the electric field. Unwanted electrochemical reactions, electrokinetic effects, and/or gas formation due to electrolysis at the tip or sample are the consequence and prevent conventional KPFM from working in water.^9^

This problem has been addressed in a number of ways, for example, by not biasing the tip and by performing AFM force–distance curves instead, where the tip is vertically moved toward and away from the sample while holding its horizontal location constant. These force-vs-distance curves contain all the contributions to the force between tip and sample at that location, mainly van-der-Waals forces and electrostatic forces. By using models based on DLVO theory, knowledge of the ionic strength of the medium between tip and sample, and—where possible—comparison with a sample of known surface charge, the unknown charge of samples such as bacterial membranes, DNA molecules or viruses was determined.^10−12^ While not actually presented in the literature, such force–distance curves and their electrostatic analysis could, in principle, be performed repeatedly on a multitude of locations across a surface to obtain maps of the surface charge.

Model-independent and practically simpler methods were developed in form of open-loop KPFM (open-loop electrostatic potential microscopy (OL-EPM),^13^ dual harmonic KPFM (DH-KPFM),^14^ or general acquisition mode KPFM (G-mode KPFM))^15^ which omit the use of a DC-bias and, thus, the feedback loop that nullifies the above-mentioned electrostatic force between tip and sample. These modes were demonstrated successfully on nanoparticles functionalized with charged amino- and carboxy-groups in low-molarity NaCl solutions,^13^ by mapping the distribution of corrosion cells on stainless steel,^16^ and in profiling the electrical double layer (EDL) above a charged surface exposed to an ion-containing liquid.^17^

DH-KPFM and its derivatives^14,18^ demonstrate the fundamental feasibility of spatially resolved surface potential measurements using AFM in aqueous solutions. Because the nullifying feedback loop is omitted, the frequency-dependent cantilever dynamics needs to be known in order to obtain a quantitative measure of the surface potential. This is achieved by calibration before each measurement, where the cantilever is mechanically excited, and its amplitude- and phase-response are recorded. However, besides inevitable uncertainties of the calibration measurement, the dynamics of an AFM cantilever is very sensitive to drift (e.g., temperature) or changes of its effective mass. As in any oscillating system, a change of the effective mass leads to a change in resonance frequency (and, hence, effective amplitude if the amplitude is measured at the unchanged, original frequency). Tip wear^19^ or unwanted pick-up of material, often in measurements on fragile, biological samples,^20^ has been shown to change the effective mass, thereby leading to an unwanted alteration of the cantilever dynamics that cannot be distinguished from the desired electrostatic signal. Therefore, closed-loop approaches would still be preferable since they do not require calibration and do not rely on an unaltered cantilever dynamic. Instead, they continuously compensate for any change of the cantilever dynamical response.

We, therefore, developed an alternative method, which we termed AC-KPFM.^21^ The method keeps the closed-loop compensation principle of classical KPFM and equally operates as a two-pass scan technique, where, after the topography measurement, the surface potential, φ, is mapped. In KPFM, the electrostatic force acting on the cantilever is modulated with the application of an AC-voltage between tip and sample. While conventional KPFM uses a DC-bias for the nullification of the cantilever deflection caused by this force, AC-KPFM uses a second AC voltage of twice the frequency, *U*_C_ = *a*·sin(*ωt*) + *b*·cos(2*ωt*) (Figure 1a). By controlling the amplitude *b*, the condition of zero cantilever deflection at the frequency ω (*F*_ω_ = 0) is fulfilled, leading to the localized surface potential φ = *b*/2. Since no DC biases are involved in AC-KPFM, parasitic electrochemical events are prevented, and operation in aqueous environments is feasible as the above-mentioned open-loop techniques show.

**Figure 1:**
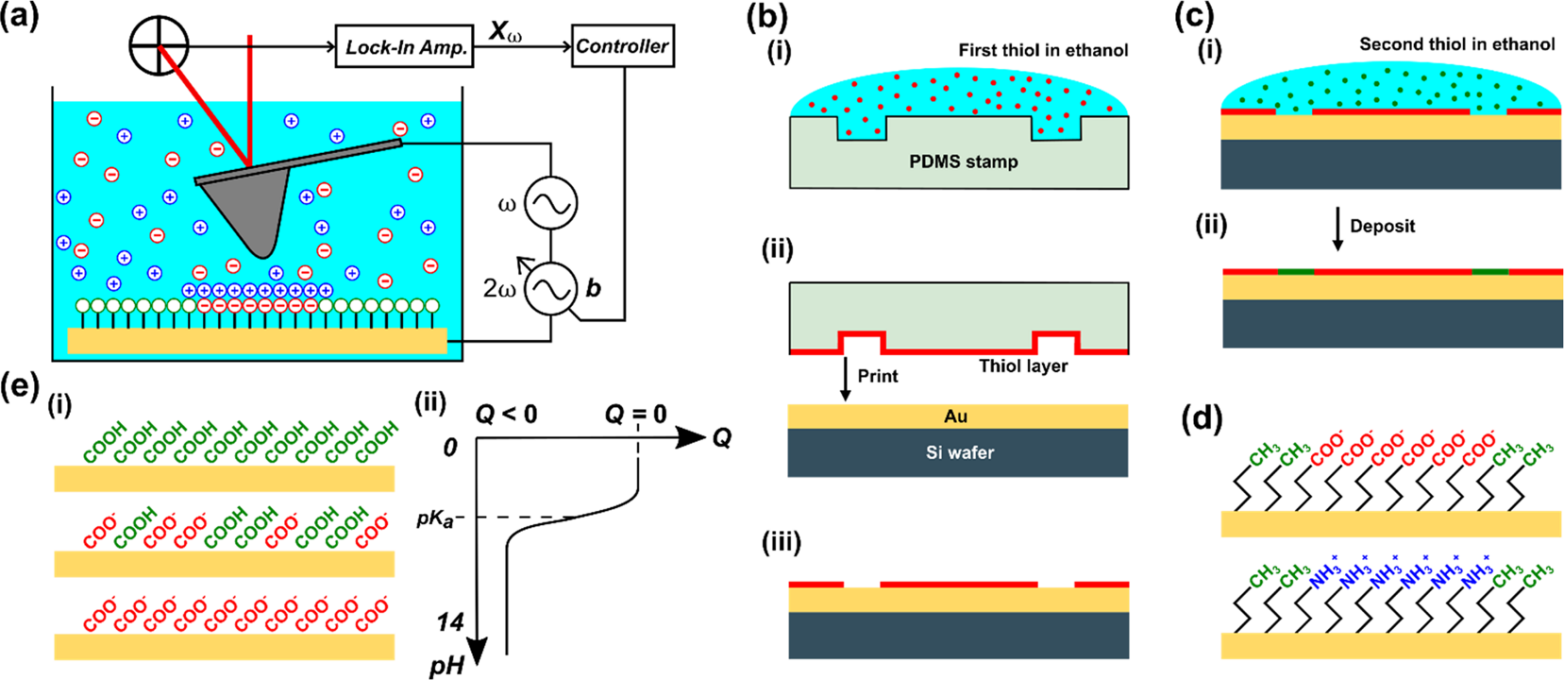
AC-KPFM of microcontact-printed SAMs. (a) AC-KPFM measurement in aqueous solution with anions and cations. The cantilever deflection amplitude *X*_ω_ arising from the electrostatic force is nullified by controlling the amplitude *b*. (b) A first thiol, e.g., with carboxy-(COOH)-functionalized groups, is microcontact-printed on a bare Au surface using a polydimethylsiloxane (PDMS) stamp (i–iii). (c) A second thiol functionalized with methyl-(CH_3_)-groups fills the unoccupied areas (i–ii), forming a self-assembled monolayer. Residuals are removed by rinsing with pure ethanol. (d) Resulting, ideal arrangement of negative (COO^–^), positive (NH_3_^+^), and uncharged groups (CH_3_) on the surface. (e) Adjusting of the ionization state of surface groups (i) by changing the pH of the solution (ii). At low (acidic) pH ≈ 0, most carboxy groups are protonated, and, hence, the overall surface charge is near zero (*Q* ≈ 0). At high (basic) pH ≈ 14, most carboxy groups are deprotonated, and, hence, the overall surface charge is maximally negative (*Q* < 0). The inflection point of the *Q*-vs-pH curve of this particular arrangement of surface groups can be defined as the effective p*K*_a_ of these surface groups.

In the present paper, we demonstrate the ability of AC-KPFM to perform quantitative surface potential measurements in low-molarity aqueous solutions (in deionized water and in solutions between 5 mM and 20 mM) and compare them with measurements in air. An important aspect is to ascertain the true, electrostatic nature of the signal, independent of the surface topography. We, therefore, used microcontact-printed, amino-(NH_2_)- and carboxy-(COOH)-functionalized self-assembled monolayers (SAMs) of alkanethiols on flat gold surfaces, thereby minimizing the influence of the surface topography (Figure 1b–d). The ionizable, chemical groups on the surface act like spatially fixed charges, whose magnitude can be adjusted by varying the pH of the solution (Figure 1e). These chemical groups are, in fact, representative of the most relevant, ionizable groups in biomolecules as they occur in several amino acids and, hence, in all proteins. Moreover, the patterns have characteristic sizes of tens of μm. This allows us to, first, fit them within the scan range of an AFM image, and, second, to minimize edge effects caused by the limited lateral resolution of the KPFM measurement.

## Results and Discussion

### AC-KPFM in Water Compared to Air

**Figure 2:**
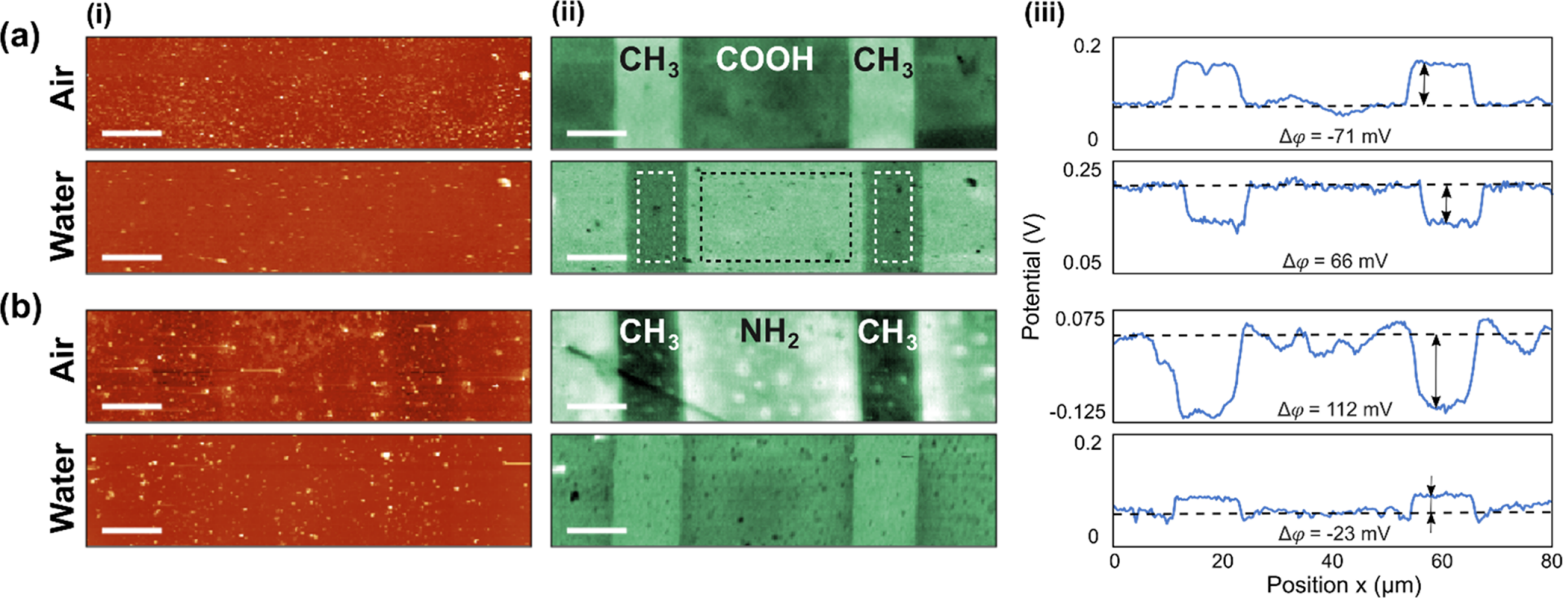
Examples of AC-KPFM images of micropatterned SAMs. Topography (column (i)) and surface potential determined by AC-KPFM (column (ii)) with representative cross sections and measured potential differences (column (iii)). (a) COOH/CH_3_ pattern and (b) NH_2_/CH_3_ pattern, in air and deionized water as indicated. The potential values inside the dashed rectangles are used in the statistical analysis. (Scale bar = 10 μm, topography color range = 100 nm, potential color range = 200 mV).

Figure 2 shows an example of AFM topography (column (i)) and surface potential measurements by AC-KPFM (column (ii)) of micropatterned SAMs of CH_3_/COOH (row (a)) and CH_3_/NH_2_ (row (b)) terminated alkanethiols in air and in deionized water. As the SAMs have approximately equal height, they are not prominently visible in the topography images. In the potential maps (ii), however, a clear contrast between the differently ionized SAMs is visible. This demonstrates that the signal acquired by AC-KPFM is not a topography or edge artifact. As usual in electrical AFM modes, the potential maps, both in air and water, show relative quantities. For example, in the map (a)(ii) in air, the potential of the COOH-terminated SAM appears ca. 70 mV lower, that is, more negative than the CH_3_-terminated SAM.

The potential maps taken in air show the same relative polarities as in our earlier works,^7^ and they are commensurate with KPFM measurements in air of other structures made by ourselves^22−25^ and others.^6,13,26−29^ In ambient air, the samples are still covered with a thin layer of water, which permits proton donation and acceptance and, thus, ionization. In terms of their chemistry, COOH groups can only be ionized negatively as COO^–^ groups by donating a proton, and NH_2_ groups can only be ionized positively as NH_3_^+^ groups by accepting a proton (Figure 1d). As the groups are effectively fixated on the samples, their charge is, therefore, seen in the potential maps in air.

When AC-KPFM is performed in deionized water in the same locations of the samples as performed in air, a sign reversal of the potential is observed (Figure 2(ii)). COOH regions appear more positive, and NH_2_ regions appear more negative than CH_3_ regions, respectively. The ionization chemistry of these groups cannot change to the extent of complete polarity reversal. An altered potential of the CH_3_ groups also cannot explain this behavior as it would affect both samples in the same way. So, how can the sign reversal of the potential images in water be explained?

The most likely cause is the adsorption of a very thin layer of counterions from the water (Figure 1a). The ionized thiols exhibit a strong surface charge attracting oppositely charged, mobile ions from the solution (blue, positive ions in the example of Figure 1a). These ions form the first countercharge layer of the EDL at the interface between water and the immobile alkanethiol SAM, that is, the Stern layer.^1^ In other words, we posit that it is the charge of the Stern layer which is measured in AC-KPFM in water, not the actual charge of the fixated thiol groups. This hypothesis will be investigated further below.

Another observation concerns the magnitude of the AC-KPFM signal of a given sample in water compared to air. For example, Figure 2(a)(iii) shows that the potential contrast of COOH vs CH_3_ is approximately 70 mV in air. In water, the contrast is similar. However, Figure 2(b)(iii) shows that the potential contrast of NH_2_ vs CH_3_ is approximately 110 mV in air but only 20 mV in water. The most likely explanation for this discrepancy is that it is not always possible to map exactly the same area of the sample when water is removed or added. The position of the tip could move by a few tens of μm when filling the sample cell with water. As the stamping process does not always produce exactly the same density of SAMs on the substrate, the potential contrast could simply vary considerably along the stripes.

### Dependence of AC-KPFM Signal on pH

**Figure 3:**
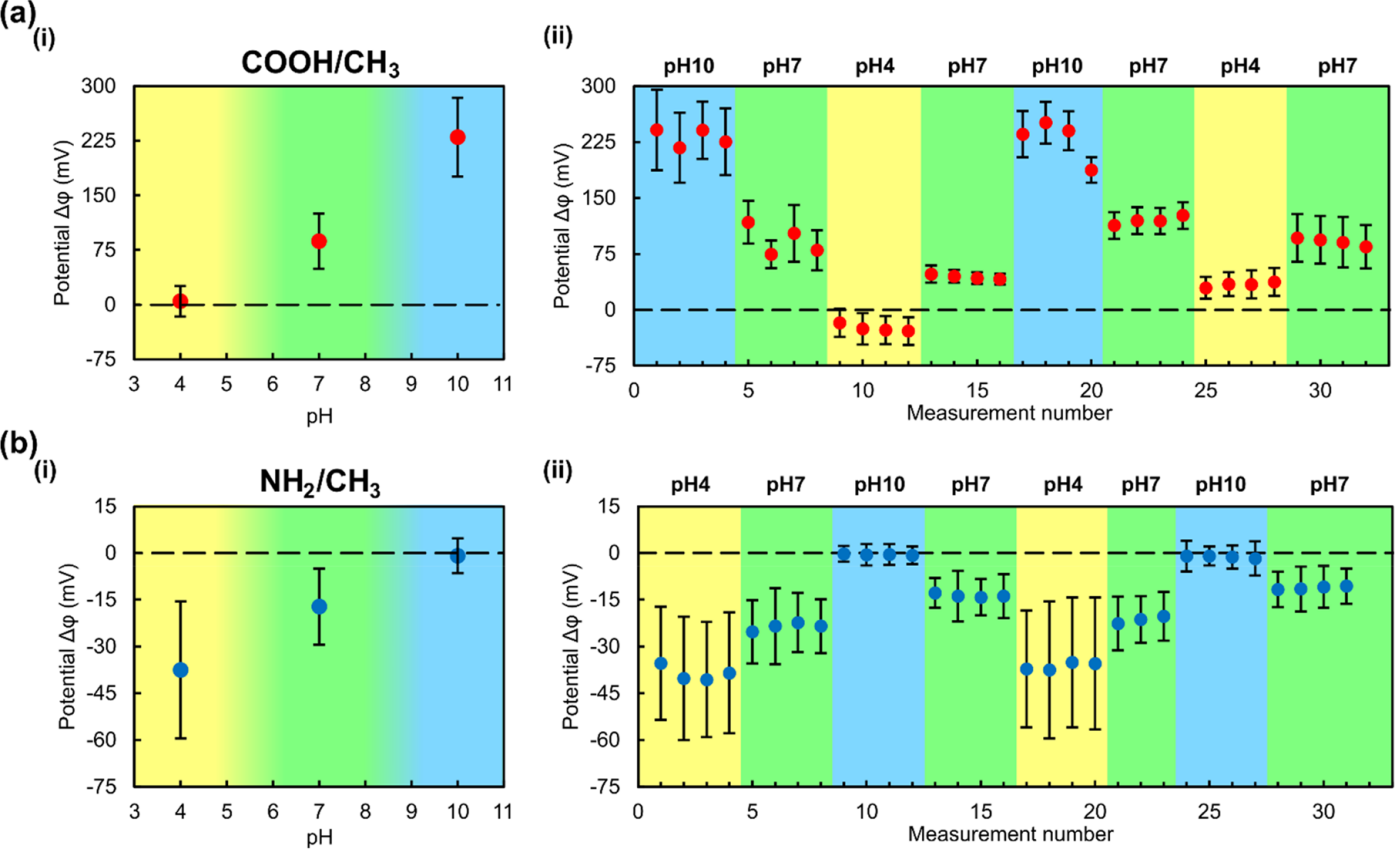
Dependence of surface potential on pH of the solution. The surface potential shown is the difference of the measured potential of the COOH or NH_2_ region and the CH_3_ region, respectively, (a) Δφ = φ_COOH_-φ_CH3_ or (b) Δφ = φ_NH2_-φ_CH3_. Averaged surface potential (i) of the respective pH in the measurement series (ii). The error bars indicate the maximum RMS roughness values of the respective potential maps.

In order to ascertain further the origin of the AC-KPFM signals observed in Figure 2 in water, we need to alter the surface charge displayed by the SAMs in a controlled manner. This can be performed by changing the pH of the solution because pH influences the concentration of protons in the solution and, hence, the equilibrium of the ionization of the solution-exposed groups (example of COOH/COO- ionization in Figure 1e). To this end, AC-KPFM measurements were performed in water at low (pH 4), neutral (pH 7), and high pH (pH 10) with the same samples (Figure 3). The expected behavior of COOH and NH_2_ groups is that their charge becomes more negative (Figure 1e(ii)) when the pH is changed from low to high. Due to the Stern-layer effect mentioned above, the opposite behavior would be expected in the AC-KPFM signal. That is, AC-KPFM would show an increase of the surface potential in water with pH value toward a relatively more positive potential.

This is, indeed, observed in our experiments (Figure 3a,b). For, both, COOH and NH_2_ terminated SAMs, an increase in potential is observed. The potential values are always relative to the potential of the CH_3_ region, which can be assumed to be constant as CH_3_ groups are not ionizable. Figure 3ab also shows that the potential of the COOH region is higher (= more positive) than the potential of the NH_2_ region for all pH values. Taking into account the Stern-layer effect, this means that the COOH region is, at all pH values, more negatively charged than the NH_2_ region, which is expected from the respective chemical properties of these groups. The protonation reaction of the surface-fixated groups (COOH ↔ COO^–^ + H^+^ or NH_3_^+^ ↔ NH_2_ + H^+^, respectively) is an equilibrium reaction. Increasing the pH (= making the medium more basic) means reducing the concentration of H^+^ in the medium and, thus, shifting the equilibrium to the right in both cases. That is, in either case, the surface becomes more negative when increasing the pH. The qualitative behavior is illustrated in the curve in Figure 1(e)(ii).

In order to check that the results observed in Figure 3a,b are not just an effect of drifting of the potential signals or similar artifacts caused by repeatedly scanning and reimmersing samples in water, we performed repeated and cyclical measurements shown in Figure 3a(ii) and 3b(ii). Each data point represents a full, recorded map of the CH_3_/COOH and CH_3_/NH_2_ sample, respectively. To avoid the influence of any spatial variation of the thiol surface concentration, the same area of the sample was mapped.

In AC-KPFM, as in any other lift-mode AFM measurement, the tip is brought into direct, mechanical contact with the sample many times during the topography scans (Figure 2, column (i)). This could alter the measured surface potential because the tip could remove excess material or poorly attached molecules from the surface. To check this, four consecutive maps were measured without withdrawing the tip or changing the solution for each pH value (groups of four data points each in Figure 3, with the exception of the third immersion of the CH_3_/NH_2_ sample at pH7 (measurement numbers 21–23 in Figure 3b(ii)), which had only three data points due to an accidentally leaking cell. Here, the measurement had to be stopped to prevent damage to the scanner. After removal of excess solution, the measurement was continued with the immersion in the next solution (pH10)). For most groups, the variation of the potential in consecutive maps without changing the solution was not significant, indicating stable AC-KPFM measurements with little damage of the SAMs by the tip.

Figure 3a(ii) and 3b(ii) shows that the surface potential of the SAMs is shifted according to the expected ionization behavior when changing the pH (Figure 1e(ii)) and that the shift is reversible. Both samples show a more positive signal at higher pH, which translates to a more negative surface charge of the thiols. Similarly, both samples show a more negative signal at low pH, translating into a more positive surface charge of the thiols. While these shifts are significant and unambiguous relative to each other, the potential values for a specific pH value are not well-defined, however. For example, the potentials at pH 7 for the CH_3_/COOH sample vary strongly among each other (Figure 3a(ii), green pH7 data groups). There could be many reasons for this behavior, ranging from removal or adsorption of material upon reimmersion to rearrangement of the ionizable groups. Also, the solutions were not buffered. Residual contamination of the previous solution in the liquid cell could have influenced the pH. This does not change the fundamental interpretation, however, that the charge of the ionizable groups is changed by the pH of the solution in the way known from fundamental acid–base chemistry, and that this charge alteration can be measured by AC-KPFM in water. Since the measured potential difference of the CH_3_/NH_2_ sample is rather small (∼30 mV), the values of the pH 4 and pH 7 series are within the error bars. However, a trend toward more positive values when increasing the pH is observable. Here, further developments of the AFM cantilever design and read-out electronics are necessary to reduce the noise level and improve the quality of the measurement.

### Influence of Lift Height on Measured Surface Potential

**Figure 4:**
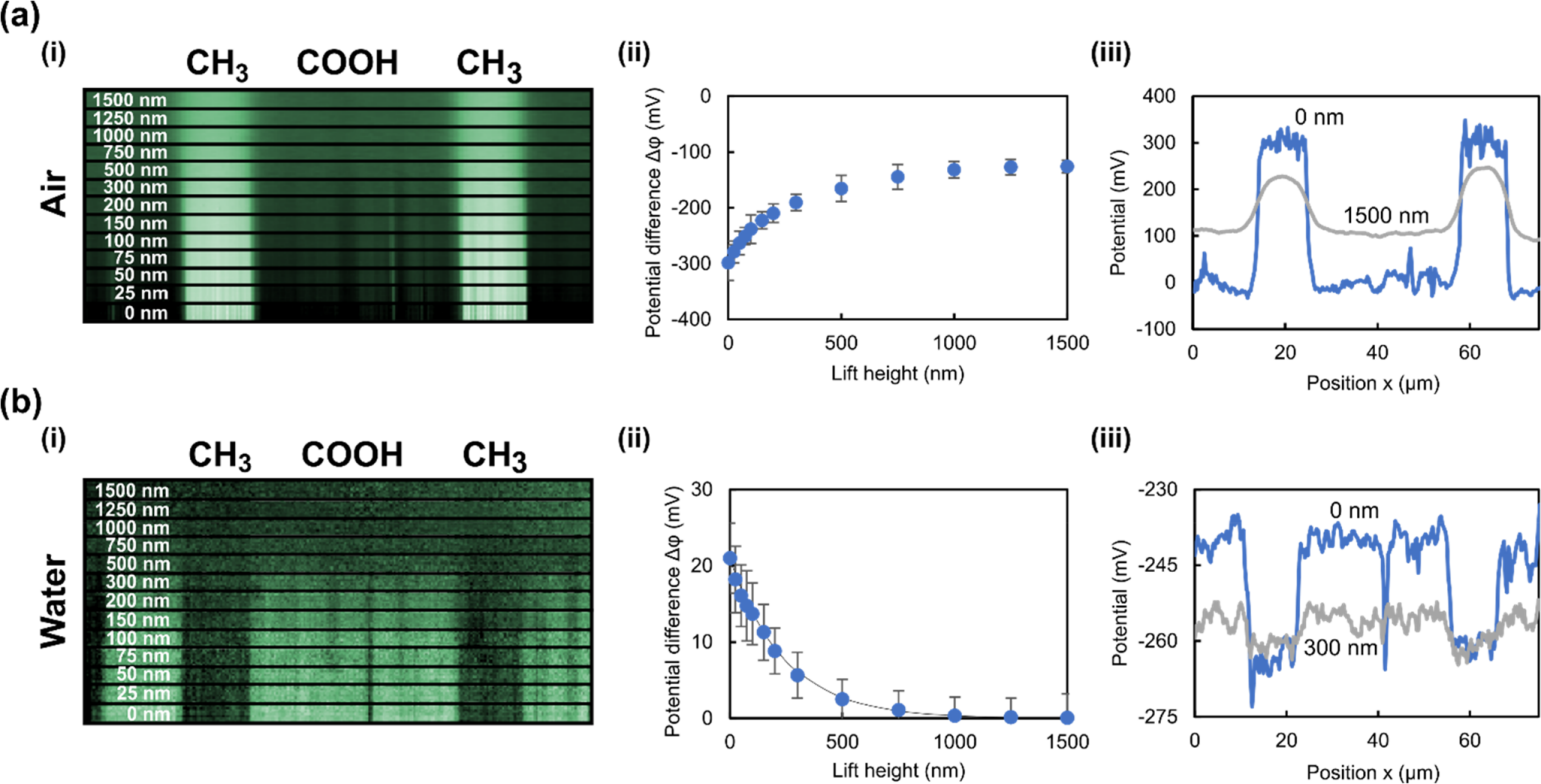
Effect of tip–sample separation. Surface potential measured by AC-KPFM on a COOH/CH_3_ sample in air (a) and deionized water (b) for different lift heights as indicated. Measured potential distribution (i), extracted potential differences Δφ = φ_COOH_-φ_CH3_ (ii), and representative cross sections at given lift heights (iii).

Figure 4a,b shows the influence of the lift height, that is, the tip–sample distance, on the surface potential measured by AC-KPFM in air (a) and deionized water (b), respectively. The sample is a micropattern of CH_3_/COOH terminated SAMs. While the AC-KPFM signal—as in classical KPFM—does not depend on the lift height in principle, spatial patterns such as the stripes used here exhibit a loss of lateral resolution upon increasing lift height. This is because a larger area of the sample contributes to the electrostatic force on the tip when it is further away (tip convolution effect).^30^ This is valid, both in air and in any other medium such as water. Figure 4a(i) was recorded in air and shows how the stripes are “smeared out” and contrast is lost with increasing lift height. Figure 4a(ii) shows that, even at the largest possible lift height of 1.5 μm, a contrast of ca. −120 mV could be detected.

In deionized water, the lift height dependence is similar but much more pronounced (Figure 4b). The tip convolution effect is also present, but decay of the potential contrast is greater than in air. The potential difference decreases exponentially with increasing lift height (Figure 4b(ii)) and becomes unmeasurable for lift heights greater than approximately 1 μm. This exponential decrease can be attributed to the influence of the electrical double layer above the charged surface and the exponentially decaying ion concentration near the surface.^17^ Although the measurement was performed in deionized water, a small number of residual, solvated ions is always present in the solution, coming from possible contaminations inside the liquid cell and from the self-dissociation of water itself. The fact that the signal decays faster with increasing lift height also supports the notion that, in water, a much smaller area at the very apex of the tip contributes to the tip–sample force because any surface charges on the sample are shielded at distances greater than a few 100 nm.

### V-Curve in Deionized Water

**Figure 5:**
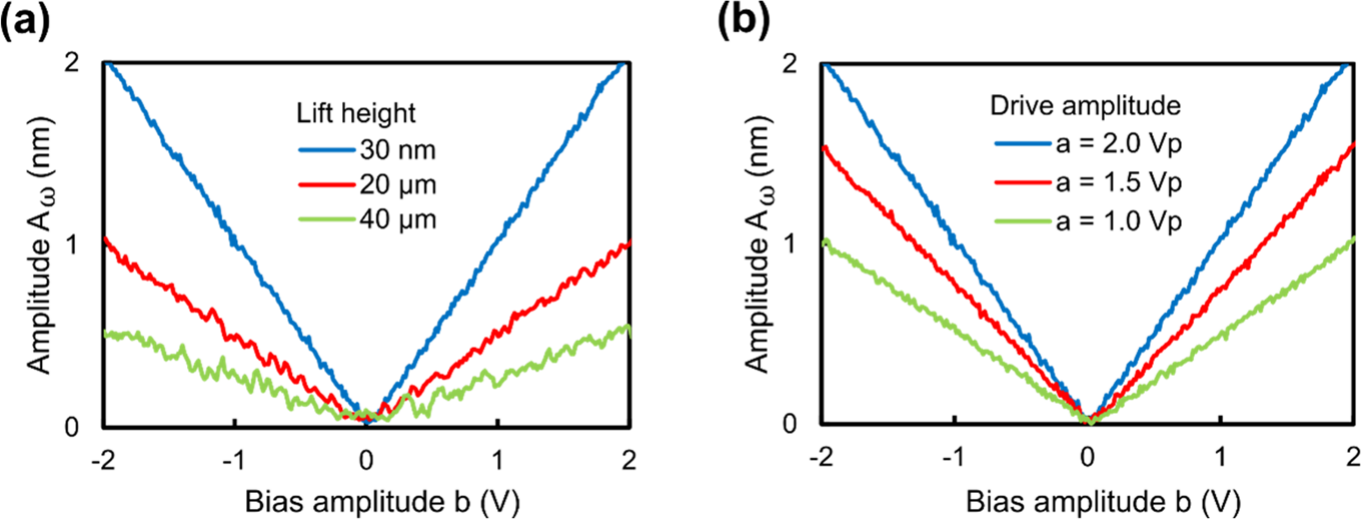
V-curve in deionized water. Open-loop cantilever deflection amplitude *A*_ω_ as a function of bias amplitude, *b*, lift height (a) (*a* = 2 Vp) and drive amplitude, *a* (b) (lift height = 30 nm). The V-curve demonstrates stable closed-loop AC-KPFM operation with the nullification of the electrostatic force in aqueous solution, leading to the measurement of the local surface potential φ = *b*/2.

In AC-KPFM, as in classical KPFM, the veracity of the electrostatic interaction, the measurement principle, the detection sensitivity, and the applicability of a controller to perform closed-loop measurements all rely on the quality of so-called V-curves, which constitute a test of how well the electrostatic force between a biased tip and the sample can be compensated down to zero by a controller. V-curves are recorded by turning off the controller and the *x*–*y* scanning motion of the tip, and measuring the amplitude of the tip oscillation, *A*_ω_, due to the constant electrical drive signal, *a*, while sweeping the bias amplitude, *b*. Ideally, these curves should show a symmetric, linear behavior on two branches around a minimum of *A*_ω_ = 0 for a particular *b* (which can have a nonzero value). The controller then finds this minimum. V-curves were performed in Figure 5 in deionized water on a bare gold surface using a gold-coated cantilever for different lift heights (a) and drive amplitudes, *a* (b). Increasing the lift height results in smaller oscillation amplitudes due to the decrease of the capacitance gradient which is proportional to the electrostatic force. However, even at very large tip-sample distances (40 μm), the V-curve preserves its desired shape, indicating correct AC-KPFM function. Furthermore, sweeping the drive amplitude, *a*, linearly adjusts the cantilever oscillation amplitude *A*_ω_ as given by the theory. These measurements, thus, show the overall correct function and capability of AC-KPFM for surface potential mapping in aqueous solutions.

### Electrostatic Adsorption of Nanoparticles

**Figure 6:**
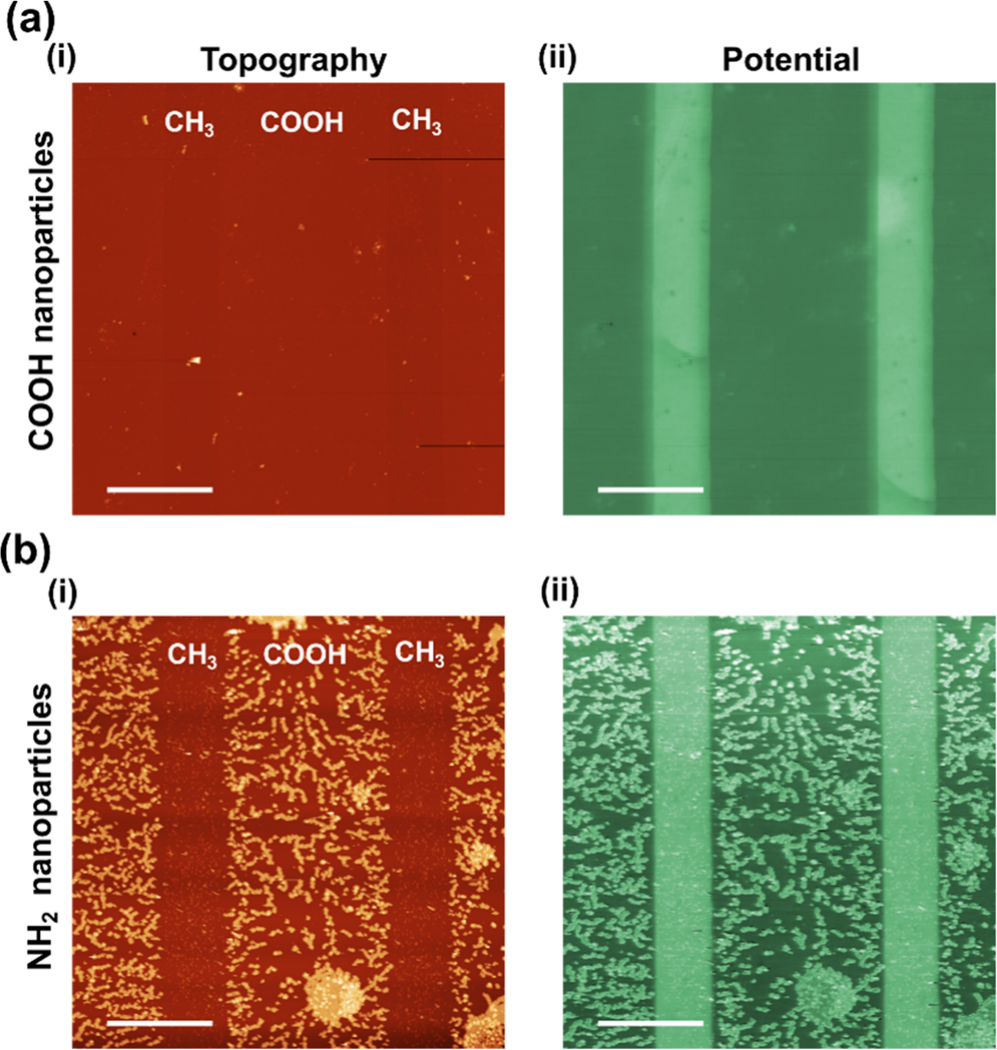
Electrostatic adsorption of NH_**2**_-functionalized nanoparticles. Topography (i) and surface potential (ii) measured in air after immersion of the CH_3_/COOH sample in a suspension of 100 nm diameter carboxy- (a) and amino- (b) functionalized latex nanoparticles and subsequent drying (scale bar = 20 μm, topography color scale range = 400 nm, potential color scale range = 500 mV). The measurement is performed in air since it was observed that, in water, the nanoparticles are pushed around by the AFM tip during the imaging.

As an additional, KPFM-independent test to confirm that the SAM micropatterns consist of flat, charged regions, we immersed a CH_3_/COOH sample in a suspension of carboxy- and amino-functionalized latex nanoparticles. The resulting topography and potential image (Figure 6(i) and 6(ii), respectively) show a clearly preferred adsorption of the positively charged particles on the negative COOH-regions, whereas negatively charged particles do not bind to the COOH surface, confirming the electrostatic nature of the surface patterns and their interaction with oppositely charged particles.

The fundamental strength of AC-KPFM is the closed-loop compensation principle. Compensation-based measurement methods have inherent advantages in that they are less prone to sudden or gradual changes of the conditions in which the measurement is made. Drifting of the cantilever dynamics is one example. As in classical KPFM, the very principle of AC-KPFM ensures that only the electrostatic force between tip and sample is nullified, and, hence, true potential values are determined. In terms of practical implementation, a significant advantage is that AC-KPFM does not require newly developed and untested hardware. Specifically, the method does not require new types of AFM tips, scanners, or similar components. As long as various signals are externally accessible, it can be implemented on most commercial AFM instruments using off-the-shelf electronic devices such as external lock-in amplifiers.

Collins *et al.* showed that, the greater the ion concentration (molarity), the higher the frequency, ω, necessary to perform the measurements.^31^ Measurements in solutions of physiological molarity (≈150 mM) would require cantilever resonance frequencies in the MHz range,^31^ which is currently not possible with commercially available standard cantilevers. New, high-resonance frequency cantilevers would need to be developed, which has been done for other reasons for many years but still faces considerable technical and practical difficulties. Alternatively, a sequence of measurements at different, low molarities (on the order of 10 mM) could be performed as far as possible, and then extrapolated to higher molarities. While not ideal, it is still conceivable that careful experimental design with tightly controlled ionic strength and pH could still lead to meaningful data, for example, on biomolecules such as DNA or proteins. The structure of such biomolecules depends more on the pH than on the ionic strength. For example, it is pH that determines the charge of protein residues and, hence, protein structure. The pH, however, can be adjusted and maintained at around 7 in an AFM measurement without the need for a buffer solution because there are no actual metabolic processes that could alter the pH around a sample prepared for AFM. In any case, the correct functioning of AC-KPFM can always be checked quickly by performing V-curve measurements.

Although not the scope of this work, it is worth considering the implications of KPFM mapping in water on spatial resolution. This could be relevant especially for mapping single biomolecules such as DNA or proteins, as these constitute very small structures by KPFM standards.^32^ KPFM fundamentally relies on electrostatic interaction, which is a long-range force. This means that a much larger part of the AFM tip and even the cantilever itself contribute to the signal, and, consequently, the spatial resolution of AM-KPFM is much worse than the topography resolution of AFM with the same tip.^4^ As there is no fundamental difference between AC-KPFM and classical AM-KPFM in terms of the tip–sample interaction, the spatial resolution is expected to be generally comparable. However, our AC-KPFM measurements in water (Figure 4) show a faster drop-off of the signal upon increasing lift height compared to air. This, in turn, means that tip and cantilever parts further away from the sample interact less with the sample in water than in air. These far-away parts do not “see” the surface charge in water as much as they would in air. A plausible interpretation is that screening of surface charges by the EDL is the cause. Typical screening distances are quantified by the Debye length, which depends on the ionic strength of the solution and which is broadly in the range of 1 nm to 1 μm (the greater the ionic strength, the smaller the Debye length). The tip itself has a height of several tens of μm and, therefore, reaches well outside the screening distance. One can, thus, expect an improvement of the spatial resolution of KPFM measurements in water compared to air because a smaller part of the tip interacts with the sample. However, there is a trade-off. Because of the overall smaller interaction force between tip and sample, the SNR is also expected to be smaller. A more comprehensive study of these competing effects, for example, using well-defined biomolecules such as DNA as test structures, would be useful in the future. From a more fundamental point-of-view, it would also be useful to investigate more systematically how the measured AC-KPFM surface potential relates to the various potentials defined in DLVO theory, such as the potential of the Stern layer, the zeta-potential, etc. For example, is the AC-KPFM potential simply the inverse of the zeta-potential that would be expected in more traditional experiments based on dynamic light scattering? Test structures based on well-defined, functionalized nanoparticles, whose zeta-potential is known and can always be verified, could be used for this purpose.

## Conclusion

In this paper, we presented the application of AC-KPFM in water, by measuring the potential distribution of charged alkanethiol layers. We investigated the influence of the pH value on the ionization state, discussed the impact of the electric double layer on the AC-KPFM signal, and showed closed-loop KPFM images taken in low-molarity aqueous solutions. Until now, the lack of such a capability has certainly limited the usage of KPFM in biological applications, which contrasts with the otherwise decades-long success of KPFM in the materials and semiconductor sciences. Many applications in biology or related fields are conceivable. For example, there is evidence that the surface charge profile of extracellular matrix fibers such as collagen is altered in glycation, which is an unwanted and uncontrolled consequence of long-term exposure of proteins to sugars. The altered surface charge could affect the interaction of collagen with cell adhesion proteins, which—in turn—could affect cell adhesion, cell motility, and, possibly, cell differentiation.^24,33^ Another example is histone acetylation, where amino groups of lysines are acetylated, thereby reducing the positive charge of the histone, which then affects its interaction with DNA in the very fundamental process of DNA packaging in the cell cycle. In fact, acetylation of lysines has now been recognized as one of the most fundamental post-translational modifications of many non-histone proteins, affecting myriads of phenomena in disease from gene regulation to cell signaling.^34^ Such modifications are important potential targets for pharmacological interventions.

## Methods

### Sample and Solution Preparation

Polydimethylsiloxane PDMS stamps for microcontact printing were prepared by casting a 10:1 mixture of polydimethylsiloxane and curing agent Sylgard 184 (Dow Corning, USA) on a patterned silicon master, which was then left for 48 h to cure at room temperature.^35^ The patterned section of the stamp was cut out with a scalpel and peeled off from the master. The relief structure of the silicon master, which was fabricated by standard photolithography, consisted of many parallel lines with a width of 10 μm and height of 5 μm. The lines were separated by 30 μm. For the alkanethiol solutions, three differently terminated alkanethiols were used: mercaptohexadecanoic acid HS-(CH_2_)_15_-COOH, 16-amino-1-hexadecanethiol hydrochloride HS-(CH_2_)_16_-NH_2_, and hexadecanethiol HS-(CH_2_)_15_-CH_3_ (all from Merck, Germany). The solutions were prepared by dissolving the thiols in pure ethanol (0.5 mg/mL).

### Substrate Fabrication, Stamp Inking, and Microcontact Printing

A p-doped silicon wafer coated with a 50 nm gold film, fabricated by e-beam evaporation (Micro-To-Nano, Netherlands), was cut into 5 mm × 5 mm size pieces, cleaned, and glued on a steel AFM specimen disc using conductive silver paint (Micro-To-Nano, Netherlands). The Au/Si substrates were cleaned by putting them into acetone, isopropyl alcohol, and deionized water, each for 5 min in an ultrasonic bath. Inking of the PDMS stamp was performed by placing a few drops of alkanethiol solution onto its patterned surface for 30 s (Figure 1b(i)). The stamp was then dried using a manually operated bellows air blower. Immediately after inking, the stamp was manually placed onto the gold surface (ii). The adhesive force between the inked PDMS stamp and the gold surface, together with a small amount of manual pressure, was sufficient to ensure conformal and stable contact during the printing. After 5 s, the stamp was manually removed, leaving the patterned alkanethiols on the surface (iii). To backfill all the unoccupied areas of the sample, a drop of a different alkanethiol solution was placed on the gold surface (Figure 1c(i)). After 30 s, the sample was rinsed with pure ethanol and dried using the manual air blower (ii). As illustrated in Figure 1d, two samples featuring CH_3_/COOH and CH_3_/NH_2_ alkanethiols were prepared.

### Aqueous Solutions

For the surface potential measurements in water, three unbuffered aqueous solutions with pH values of 4, 7, and 10, respectively, were prepared. For acidic solutions, a few drops (≈50 μL) of acetic acid (CH_3_COOH, 99.7%, Sigma-Aldrich, USA) were added to 50 mL of highly deionized water (milli-Q water, Merck Millipore, USA) until the solution had reached pH 4. For basic solutions, the same method was used with an ammonia solution (NH_3_, 9.5%, Meffert AG, Germany) until pH 10 was reached. The pH values were always double-checked using pH indicator paper. While less accurate than an electronic pH meter, the large steps in pH from 4, over 7–10 is well above the uncertainty of the pH indicator paper.

### Nanoparticles

Carboxy- (01-02-102, Micromod, Germany) and amino-functionalized (01-01-102, Micromod, Germany), 100 nm-diameter latex nanoparticle suspensions were diluted in deionized water to a concentration of 2.5 mg/mL. A drop of the diluted suspension was then put on the sample and left for 5 min. The sample was then rinsed with deionized water and dried by a manual air blower. AC-KPFM was then performed in air on these nanoparticle-coated samples.

### AC-KPFM

The surface potential of the patterned samples was mapped by AC-KPFM in the aqueous solutions of pH 4, 7, and 10. For comparison, the same samples were also mapped by AC-KPFM in air. The AC-KPFM principle is similar to classical AM-KPFM and described in our earlier paper.^21^ Briefly, in AC-KPFM, the voltage *U*_C_ = *a*·sin(*ωt*) + *b*·cos(2*ωt*) is applied between cantilever and sample during the lift scan, which results in electrostatic force components at DC, and frequencies ω, 2ω, 3ω, and 4ω. The ω-component  is nullified by the feedback controller, which adjusts the amplitude *b* of *U*_C_ until the ω-component of the cantilever oscillation vanishes (*A*_ω_ = 0). The surface potential to be determined is then φ = *b*/2. The ω-component *A*_ω_ is obtained by demodulating the cantilever deflection amplitude with a lock-in amplifier. The in-phase component of *A*_ω_ (*X*_ω_) is used as the controller input. As in classical KPFM, the best signal-to-noise ratio (SNR) is achieved by setting ω to the resonance frequency of the cantilever and by an optimum choice of the lock-in reference phase.^36^ Additionally, when operating in aqueous solutions, the driving frequency ω should be high enough such that redistribution and movement of ions in response to the electric field (i.e., electromigration), and the resulting parasitic forces on the cantilever are suppressed,^9^ which is analyzed in more detail in the literature.^31^ Here, defined measurement conditions with the suppression of ionic motion and charge dynamics are ensured by keeping the ionic concentration of the solutions low, the use of cantilevers with a high enough resonance frequency in water (ca. 110 kHz), and by continuous checks of the V-curve (Figure 5) throughout the measurements guaranteeing correct AC-KPFM operation.

AC-KPFM was performed on a Multimode 8 AFM (Bruker, USA) with a Nanoscope V controller and the Bruker Signal Access Module to connect the AFM with external electronics. An external signal generator (33522B, Keysight Technologies, USA) generating *U*_C_ was connected to a gold-plated cantilever (TAP300 GB-G, BudgetSensors, Bulgaria). The cantilever deflection signal was fed out of the Signal Access Module and demodulated by an external lock-in-amplifier (SR844, Stanford Research Systems, USA) by locking on the reference frequency provided by the signal generator. The in-phase component (*X*_ω_) of the demodulated signal was fed to a proportional-integral (PI) controller, implemented on a rapid prototyping system (DS1005, dSpace, Germany). The controller output adjusts the amplitude *b* to perform the above-mentioned nullification. Additionally, the signal *b* was digitized by an analog input of the Nanoscope V controller and displayed alongside the topography scan (tapping-mode) to allow the presentation of the surface potential map. Lift heights of 50 nm, scan rates of 1 line/s, drive frequencies of 110 kHz (= measured resonance frequency of the cantilever in water), and drive amplitudes of *a* = 2 V were used throughout all measurements.

### Data Analysis

All image data (see example in Figure 2) were analyzed using the open-source AFM image analysis software Gwyddion (gwyddion.net). The surface potential map was generated in a postprocessing step by dividing the recorded signal *b* by 2. The topography data were first-order line-leveled using the *align rows/median* function and then fitted by a second-order plane to remove the scanner bow (*polynomial background removal*). The surface potential data were first-order line-leveled (*align rows/median*), where regions of COOH and NH_2_ thiols were excluded from the leveling by using the *mask* function so that the CH_3_ region acts as a zero-potential reference.

The NH_2_ and COOH potential data were determined by selecting a rectangle of approximately 20 μm × 15 μm centered in the middle section of the recorded image (Figure 2a(ii)). In this rectangle, the average potential and RMS roughness were calculated using the *statistical quantities* function. The same procedure was performed for the CH_3_ potential data. Here, the rectangle (5 μm × 15 μm) was selected over the left and the right thiol stripe (Figure 2a(ii)). Some distance from the edges was maintained to exclude edge effects in the data. Dust particles on the surface, which were present in some measurements as seen in the topography image, were masked during the potential determination. The potential of the CH_3_ region (average from left and right stripe) was then subtracted from the NH_2_ or COOH region potential to determine the potential difference shown in the data points of Figures 3 and 4.
